# Axillary artery pseudoaneurysm after plate osteosynthesis for a clavicle nonunion: A case report and literature review

**DOI:** 10.4103/0973-6042.76969

**Published:** 2010

**Authors:** Gregory I. Bain, Ian J. Galley, Angus R. E. Keogh, Adam W. Durrant

**Affiliations:** Modbury Public Hospital, Modbury, South Australia; 1 Royal Adelaide Hospital, Adelaide, South Australia

**Keywords:** Clavicle, fracture, nonunion, pseudoaneurysm

## Abstract

Neurovascular complications have been reported from both plate osteosynthesis and intramedullary fixation of midshaft clavicle fractures. We wish to report a case of limb threatening ischemia from screw penetration of the axillary artery after plate osteosynthesis for a clavicle nonunion. A literature review of vascular trauma from midshaft clavicle fractures is presented.

## INTRODUCTION

Clavicle fractures are common, accounting for 5–12% of all fractures.[1,2] The results of non-operative management of middle third clavicle fractures are typically good with a rate of nonunion between 0.1 and 5%.[1,3,4] Internal fixation of clavicle fractures in patients with a high risk of nonunion has been advocated.[5–9] Appreciation of the negative effect on shoulder function of clavicle shortening,[10–12] the availability of anatomy specific locking plates and intramedullary devices has also increased the popularity of clavicle fixation.

Neurovascular complications have been reported from both plate osteosynthesis[13–15] and intramedullary fixation of midshaft clavicle fractures.[16]

We wish to report a case of limb threatening ischemia from screw penetration of the axillary artery after plate osteosynthesis for a clavicle nonunion. A literature review of vascular trauma from midshaft clavicle fractures is presented.

## CASE REPORT

A 32-year-old male carpet layer suffered a closed fracture to his nondominant left clavicle in 1998. He was treated non-operatively and went on to develop a symptomatic nonunion. Open reduction and bone grafting of the nonunion was performed in 1999 with a six-hole AO small fragment dynamic compression plate (DCP) (Synthes^®^).

The clavicle united and his symptoms resolved. He presented 6 years after his surgery, with 18 months of progressively worsening pain, paraesthesia and claudication in his left hand. In the week prior to presentation, he noticed that his left hand was cool and pale.

Clinically, he had a well-healed scar over his left clavicle with no associated mass or bruit. His left axillary artery was palpable. However, his brachial, radial and ulnar arteries were not. He had pain in the hand with exertion and poor capillary return. There were no trophic changes. His upper limb neurology was normal.

**Figure 1:**
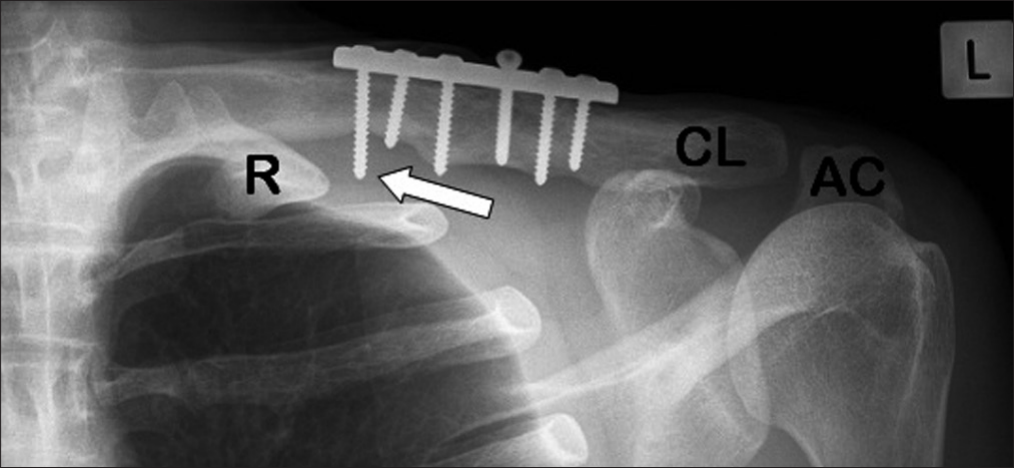
Plain radiograph of left clavicle. (AC: Acromion. CL: Clavicle. R: 1^st^ rib). Arrow depicts prominent medial screw lateral to the 1^st^ rib

Plain radiographs demonstrated a united clavicle with a six-hole plate superiorly. The medial screw was prominent inferiorly [Figure 1].

**Figure 2:**
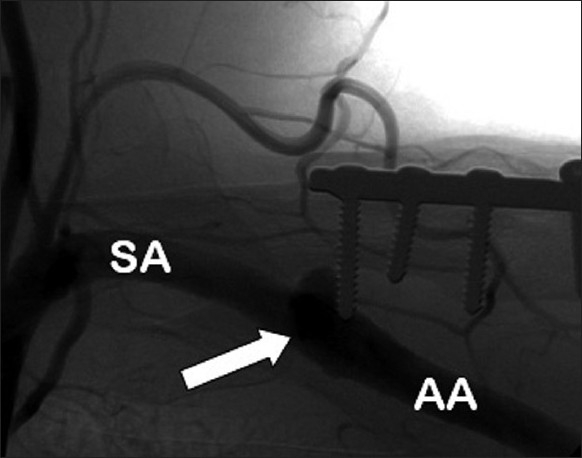
Arteriogram. AA: axillary artery. (SA: Subclavian artery). Arrow depicts penetration of the axillary artery by the prominent medial screw. A pseudoaneurysm has formed. The axillary artery is filled with contrast distal to the pseudoaneurysm

**Figure 3:**
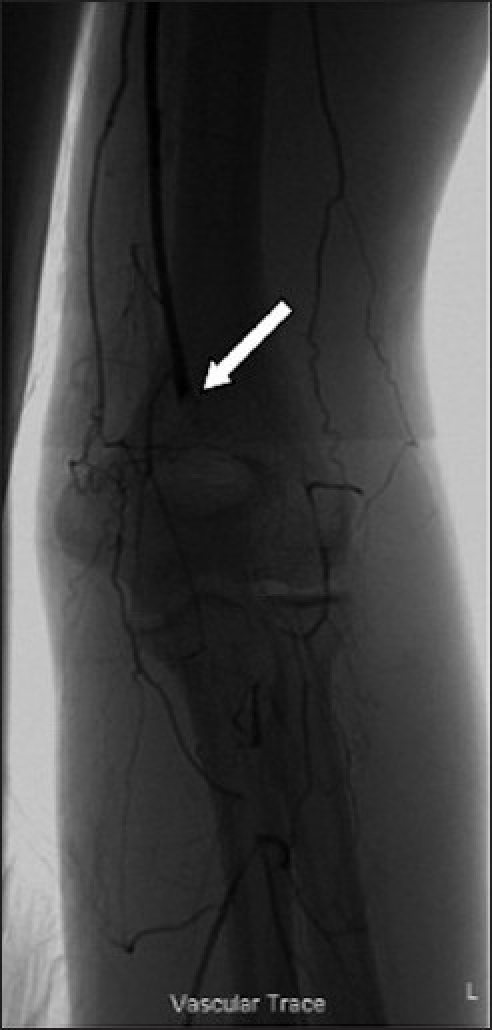
Arteriogram at level of left elbow. The arrow indicates occlusion of the brachial artery. Sluggish distal perfusion is provided via collateral vessels at the level of the elbow

**Figure 4:**
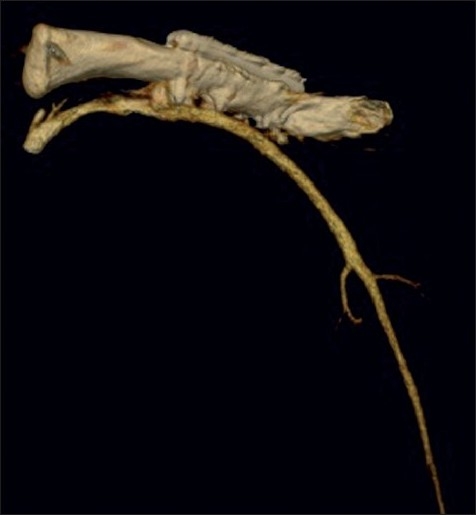
CT arteriogram of left clavicle, demonstrating penetration of the axillary artery by the prominent medial screw with associated pseudoaneurysm of the axillary artery. All structures apart from the clavicle and subclavian and axillary arteries have been subtracted

**Figure 5:**
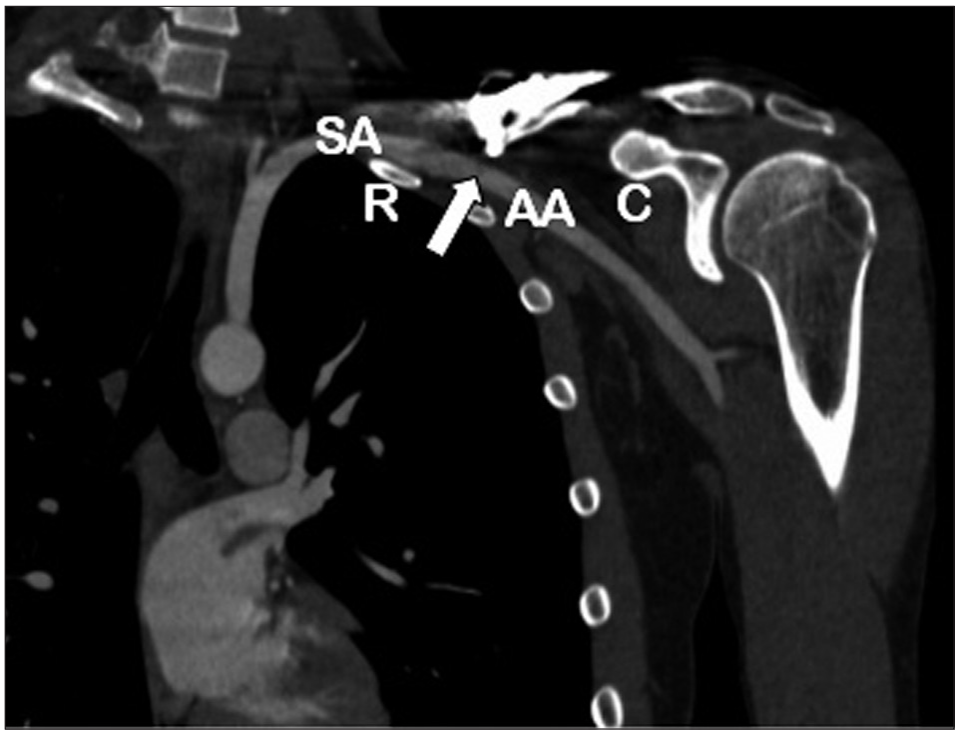
Coronal 2D reconstruction CT arteriogram of left shoulder. (AA: axillary artery; C: Coracoid process; R: 1^st^ rib; SA: Subclavian artery). Arrow depicts prominent screw with associated pseudoaneurysm. The subclavian artery becomes the axillary artery at the lateral border of the first rib. The subclavian artery lies in a groove on the superior aspect of the 1^st^ rib

An arteriogram demonstrated the medial screw penetrating the axillary artery with an associated pseudoaneurysm [Figure 2]. There was occlusion of the brachial artery above the elbow [Figure 3] with slow filling of the ulnar artery via collateral vessels. The radial artery and the proximal one-third of the ulnar artery were occluded. There was little flow past the metacarpophalangeal (MCP) joints. A computed tomography (CT) arteriogram confirmed penetration of the axillary artery by the prominent medial screw, with an associated pseudoaneurysm [Figures 4 and 5].

**Figure 6:**
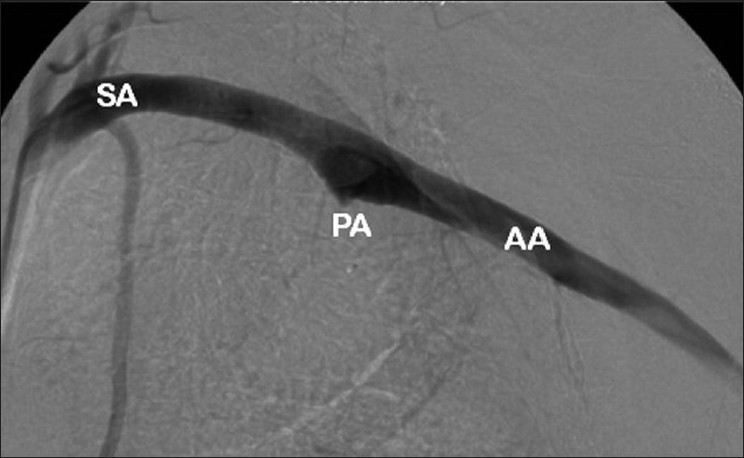
Arteriogram of left subclavian and axillary artery following removal of internal fixation. (AA: Axillary artery; PA: Pseudoaneurysm; SA: Subclavian artery)

The patient was anticoagulated following plate removal [Figure 6]. The prominent medial screw measured 26 mm.

The pseudoaneurysm was bypassed using an 8 mm by 25 mm Viabahn^®^ (W. L. Gore and Associates, Flagstaff, AZ, USA) stent placed in the axillary artery via a femoral approach. The self-expanding stent is constructed with a durable, reinforced biocompatible, expanded polytetrafluoroethylene (ePTFE) liner attached to the external nitinol stent structure.

**Figure 7:**
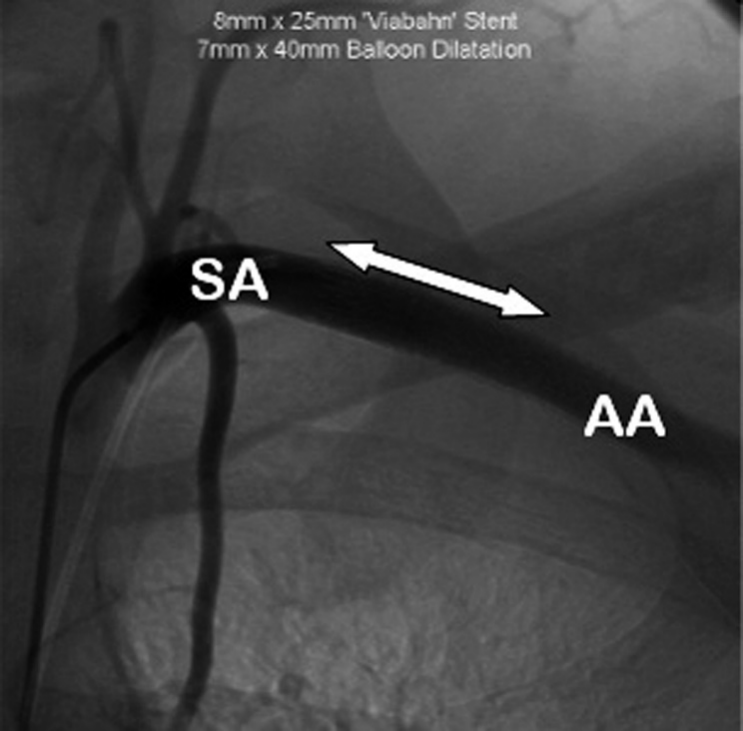
Arteriogram of left subclavian and axillary artery following insertion of 8 mm×25 mm Viabahn® (WL Gore, Flagstaff, AZ, USA) endoprosthesis, shown by arrow, after balloon dilation with 7 mm×40 mm balloon. (AA: Axillary artery; SA: Subclavian artery). The neck of the pseudoaneurysm has been occluded

The stent was dilated using a 7 mm×40 mm balloon [Figure 7].

## DISCUSSION

Iatrogenic vascular injury from internal fixation of midshaft clavicle fractures is rare, with only three other reported cases from plate osteosynthesis. Johnson[14] reported a case of left arm ischemia presenting 22 months after internal fixation of an acute fracture. A pseudoaneurysm with occlusion of the brachial artery was found. Ligation of the subclavian artery followed by carotid-axillary bypass grafting resulted in a symptom-free left arm.

Casselman[13] reported a similar case, 8 years following internal fixation of a nonunion. The pseudoaneurysm and first rib was resected via a transclavicular approach. Interposition grafting was performed using Gor-Tex (W. L. Gore and Associates, Flagstaff, AZ, USA). The clavicle was bone grafted and internally fixed at the end of the procedure.

Shackford[15] reported a case, 10 years following internal fixation of a midshaft nonunion. Angiography confirmed a pseudoaneurysm with screw penetration of the subclavian artery and occlusion of the brachial artery. Resection of the pseudoaneurysm confirmed screw penetration of the artery. Interposition vein grafting and claviculectomy gave a good clinical result.

All cases had a delayed presentation ranging from 22 months to 10 years following internal fixation. It is possible that the prominent screw erodes through the arterial wall with shoulder movement. Another explanation is that the artery is injured at the time of injury or surgery and forms a pseudoaneurysm, which was found in all cases. The upper limb has an extensive collateral circulation[17] particularly around the shoulder and elbow. This allowed all four patients to present with claudication symptoms rather than critical ischemia despite occlusion of the brachial artery.

There have been multiple reported cases of vascular injuries from midshaft clavicle fractures.[18–24] These include arterial and venous lacerations and arterial pseudoaneurysms.[25–32] Death from vascular injury secondary to clavicle fracture has been reported.[33,34] The most famous case is that of Sir Robert Peel (1788–1850) who created the police force in Great Britain. Subclavian vein thrombosis (Paget–Schroetter’s syndrome)[35–37] from clavicle fracture and pulmonary emboli from this thrombus[38] has been reported.

Nonoperative treatments for psuedoaneurysms, such as external ultrasound compression and transcatheter coiling, have been used at other sites. However, they are not suitable for the subclavian artery. Ultrasound guided thrombin injections have been successfully used for a subclavian pseudoaneurysm.[39] There is, however, a risk of occluding one of the branches supplying cerebral circulation.

Surgical treatment can be performed by means of a supraclavicular incision and can be combined with clavicular resection. Access to the proximal subclavian artery may require a median sternotomy or a thoracotomy through the third or fourth intercostal space on the left.[18,20]

Endovascular stenting, as was used in this case, has been used previously for pseudoaneurysm associated with a clavicle fracture.[29,40] It has the advantage of being minimally invasive with a remote access site. Potential problems include late stenosis and fracture of the stents. Newer flexible drug eluting stents may prevent this problem.[29]

This case reminds us of the importance of careful screw placement for osteosynthesis of the clavicle. The proximity of the clavicle and subclavian artery has previously been described[41] and it is suggested that screws for osteosynthesis of the clavicle be no longer than 18 mm in order to avoid the subclavian artery. Pseudoaneurysm of the subclavian artery in this instance was successfully treated with a closed procedure. However, there is the potential for significant morbidity.
